# A Novel Machine Learning-Based Point-Score Model as a Non-Invasive Decision-Making Tool for Identifying Infected Ascites in Patients with Hydropic Decompensated Liver Cirrhosis: A Retrospective Multicentre Study

**DOI:** 10.3390/antibiotics11111610

**Published:** 2022-11-12

**Authors:** Silvia Würstle, Alexander Hapfelmeier, Siranush Karapetyan, Fabian Studen, Andriana Isaakidou, Tillman Schneider, Roland M. Schmid, Stefan von Delius, Felix Gundling, Julian Triebelhorn, Rainer Burgkart, Andreas Obermeier, Ulrich Mayr, Stephan Heller, Sebastian Rasch, Tobias Lahmer, Fabian Geisler, Benjamin Chan, Paul E. Turner, Kathrin Rothe, Christoph D. Spinner, Jochen Schneider

**Affiliations:** 1 Department of Internal Medicine II, University Hospital rechts der Isar, School of Medicine, Technical University of Munich, 81675 Munich, Germany; 2 Department of Ecology and Evolutionary Biology, Yale University, New Haven, CT 06520, USA; 3 Institute of General Practice and Health Services Research, School of Medicine, Technical University of Munich, 81667 Munich, Germany; 4 Institute of AI and Informatics in Medicine, School of Medicine, Technical University of Munich, 81675 Munich, Germany; 5 Department of Internal Medicine II, RoMed Hospital Rosenheim, 83022 Rosenheim, Germany; 6 Department of Gastroenterology, Hepatology, and Gastrointestinal Oncology, Bogenhausen Hospital of the Munich Municipal Hospital Group, 81925 Munich, Germany; 7 Department of Internal Medicine II, Klinikum am Bruderwald, Sozialstiftung Bamberg, 96049 Bamberg, Germany; 8 Clinic of Orthopaedics and Sports Orthopaedics, School of Medicine, Technical University of Munich, 81675 Munich, Germany; 9 Program in Microbiology, Yale School of Medicine, New Haven, CT 06520, USA; 10 Institute for Medical Microbiology, Immunology and Hygiene, School of Medicine, Technical University of Munich, 81675 Munich, Germany; 11 German Centre for Infection Research (DZIF), Partner Site Munich, 81675 Munich, Germany

**Keywords:** ascites, liver cirrhosis, proton pump inhibitor, spontaneous bacterial peritonitis, secondary peritonitis

## Abstract

This study is aimed at assessing the distinctive features of patients with infected ascites and liver cirrhosis and developing a scoring system to allow for the accurate identification of patients not requiring abdominocentesis to rule out infected ascites. A total of 700 episodes of patients with decompensated liver cirrhosis undergoing abdominocentesis between 2006 and 2020 were included. Overall, 34 clinical, drug, and laboratory features were evaluated using machine learning to identify key differentiation criteria and integrate them into a point-score model. In total, 11 discriminatory features were selected using a Lasso regression model to establish a point-score model. Considering pre-test probabilities for infected ascites of 10%, 15%, and 25%, the negative and positive predictive values of the point-score model for infected ascites were 98.1%, 97.0%, 94.6% and 14.9%, 21.8%, and 34.5%, respectively. Besides the main model, a simplified model was generated, containing only features that are fast to collect, which revealed similar predictive values. Our point-score model appears to be a promising non-invasive approach to rule out infected ascites in clinical routine with high negative predictive values in patients with hydropic decompensated liver cirrhosis, but further external validation in a prospective study is needed.

## 1. Introduction

Infection of ascites is typically related to spontaneous bacterial peritonitis (SBP). The pooled prevalence of SBP worldwide is approximately 17% (CI: 13.6–21.3%) and it is the lowest in North America and highest in Africa [1]. The frequency of secondary peritonitis (SecP) is relatively low, accounting for approximately 4.5% of all peritonitis cases in patients with liver cirrhosis [2,3]. Both SBP and SecP are associated with high mortality, making diagnosis crucial for survival [2,4,5]. Following the guidelines of the European Association for the Study of the Liver (EASL), diagnostic paracentesis is the standard procedure to rule out infected ascites in patients with hydropic decompensated liver cirrhosis [5,6,7]. However, diagnostic paracentesis is inconvenient for patients, time-consuming, and in rare cases can even lead to severe life-threatening complications such as haemorrhagic shock or organ perforation [8,9,10]. Hence, a decision aid tool would be desirable to help physicians discern whether or not patients with hydropic decompensated cirrhosis need a diagnostic puncture. In this study, we attempted to apply a machine learning approach to differentiate infected (SBP and SecP) from non-infected ascites in patients with hydropic decompensated liver cirrhosis. As delayed diagnostic paracentesis in patients with infected ascites is associated with increased mortality [4], a major aim of this machine learning approach was to develop a prediction model with an excellent sensitivity for identifying patients with infected ascites.

## 2. Results

### 2.1. Baseline Characteristics

In total, 700 episodes of decompensated liver cirrhosis undergoing abdominocentesis were included in this study. Abdominocentesis was performed in 526 episodes within 48 h and 174 episodes more than 48 h after hospital admission. Group A comprised 569 episodes of elevated cell count in ascites, including 532 SBP episodes (471 patients) and 37 SecP episodes (35 patients). The control group B included 131 episodes (125 patients). The mortality was 40.2% (204/508 patients) for group A and 4.8% (6/125 patients) for group B. The median duration of hospitalizations for group A and B was 18 days (1–287) and 8 days (1–72), respectively. Table S1 summarizes all univariable analyses of the features that were subsequently used for the Random Forest and Lasso score model. Acute gastrointestinal bleeding (*p* = 0.036), hepatic encephalopathy (*p* < 0.001), fever (*p* < 0.001), or ongoing chronic alcohol abuse (*p* = 0.001) significantly increased the risk for infected ascites in the univariable analysis. Group A presented significantly higher creatinine, C-reactive protein (CRP), bilirubin, international normalized ratio (INR), leukocytes (all *p* < 0.001), and lower platelet levels in the blood (*p* = 0.034). Clinical scores (acute-on-chronic liver failure [ACLF] score, Child–Pugh score, model for end-stage liver disease with serum sodium [MELD-Na) score, and Charlson Comorbidity Index [CCI]) were significantly higher for episodes with infected ascites (all *p* < 0.001). The use of proton pump inhibitors or immunosuppressants were more frequent in group A (*p* = 0.025 and *p* < 0.001, respectively), whereas non-selective beta-blockers were administered more regularly in group B (*p* < 0.001).

### 2.2. Random Forest Importance Measures

**Figure 1:**
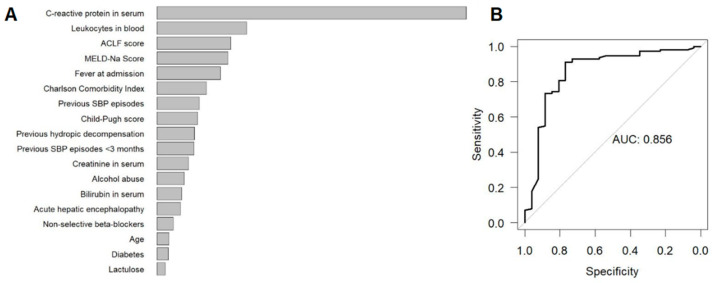
Results of the Random Forest model to identify the most important features to differentiate SBP or SecP from uninfected ascites prior to diagnostic paracentesis. (**A**) Internally validated (out-of-bag) variable importance of most important features. (**B**) Internally validated (out-of-bag) ROC and AUC of the Random Forest model. Abbreviations: ROC, receiver operating characteristics curve; AUC area under the ROC.

The most important features for differentiation between groups A and B were assessed by the Random Forest model (Figure 1A). Inflammatory markers (CRP and leukocytes), followed by markers for organ failure (ACLF/MELD-Na score) and fever were the most indicative features for differentiation. In addition, the extent of comorbidities (CCI) and the occurrence of previous hydropic decompensation (ascites) or SBP episodes were further important criteria of differentiation. The Random Forest model showed high discriminatory power in the internal validation (area under the receiver operating characteristic curve, out-of-bag AUC = 0.87, Figure 1B).

### 2.3. Scoring Model

A Lasso regression model was used to establish an additive point-score to differentiate infected ascites (SBP and SecP) from non-infected ascites. The model yielded 11 features for differentiation between groups A and B (Table 1 and [11]). Counting from zero, the probability of being classified in group A increases. By aiming at a sensitivity ≥95%, the ideal cut-off for the presence of infected ascites was determined at a score of >72. The median cross-validated sensitivity was 94.7%, implying that 5.3% of episodes with infected ascites are expected to have a score of ≤72. The median cross-validated specificity was 42.3%. Fagan’s nomograms (Figure S1) assessed the post-test probability of infected ascites (SBP and SecP). Assuming pre-test probabilities of 10%, 15%, 20%, 25%, and 30% for infected ascites (SBP and SecP) in the cohort of liver cirrhosis with hydropic decompensation, a score ≤72 leads to respective post-test probabilities of 1.9%, 3.0%, 4.2%, 5.5%, and 7.0% and negative predictive values of 98.1%, 97.0%, 95.8%, 94.6%, and 93.1%. A score >72 leads to post-test probabilities of 14.9%, 21.8%, 28.3%, 34.5%, and 40.3%, complying with the positive predictive values for infected ascites. Apart from the main score model, a simplified score model was generated, which included only those features that are easy and fast to collect in clinical routine. At a target sensitivity of ≥95%, the ideal cut off score for infected ascites, was determined at a score point of >23. For the above-mentioned prevalence rates (10%, 15%, 20%, 25%, and 30%), the negative and positive predictive values of the simplified score model were 98.1%, 97.0%, 95.9%, 94.6%, and 93.1% and 14.6%, 21.4%, 27.8%, 34.0%, and 39.8%, respectively.

## 3. Discussion

Infected ascites is one of the most life-threatening complications in patients suffering from liver cirrhosis. Therefore, early diagnosis of infected ascites is crucial for survival. However, since up to 30% of patients with SBP are asymptomatic, clinical signs such as abdominal pain or fever are not definitive for diagnosis [12,13]. To date, abdominocentesis remains the gold standard in diagnosing infected ascites, but its invasiveness, which may lead to complications such as bleeding or perforation, represents a limitation. 

In this retrospective multi-centre study, using Random Forest and Lasso models, two different statistical approaches were employed to assess the importance of 34 clinical and laboratory features for non-invasive differentiation of infected from non-infected ascites in patients with liver cirrhosis. The Random Forest model ranked the importance of all of the features, revealing that inflammatory markers (CRP and leukocytes), the presence of organ failure (ACLF/MELD-Na score), fever, the extent of underlying comorbidities (CCI), a history of ascites, and prior SBP episodes are well suited for differentiating infected from non-infected ascites. In addition, the model showed high discriminatory power in the internal validation (out-of-bag AUC = 0.87). To provide a convenient diagnostic tool for clinical practice and to accurately assess the probability of infected ascites, we used the Lasso regression based on a point-score model, which achieved a cross-validated AUC = 0.83 using 11 features. Inflammation markers, followed by patients with previous hydropic decompensation, the presence of organ failure, fever, acute gastrointestinal bleeding, and proton pump inhibitor (PPI) use were the most important features for differentiation. At pre-test probabilities of 10%, 15%, 20%, and 25% for infected ascites (SBP and SecP), a score ≤72 leads to negative predictive values of 98.1%, 97.0%, 95.8%, and 94.6%, respectively. Thus, the Lasso score point model is appropriate to rule out infected ascites. In contrast, the positive predictive value for infected ascites was relatively low. At pre-test probabilities of 10%, 15%, and 20% for infected ascites, a score >72 was associated with positive predictive values of 14.9%, 21.8%, and 28.3%, respectively. Thus, the score model is not reliable for establishing the diagnosis of infected ascites without abdominocentesis. To provide a score model with parameters that are fast and easy to collect, we established a simplified score model (Table 1). This model had a similar predictive pattern to the main score model, with a high suitability to exclude infected ascites but also a low ability to establish the diagnosis of infected ascites. 

The previous attempts at a score model to determine the risk of SBP before paracentesis included different measurements. Wehmeyer et al. identified thrombocytopenia ≤100,000 cells/µL, age > 60 years, and CRP > 6.0 mg/dL as independent risk factors for SBP in a prospective study design in Germany [14], and the respective model was validated in an Egyptian study cohort with the adjustment of CRP > 1.35 mg/dL [15]. The Egyptian ‘Mansoura simple scoring system’ combined age ≥ 55 years, mean platelet volume ≥ 8.5 fl, neutrophil-to-lymphocyte ratio ≥ 2.5, and CRP ≥ 4 mg/dL in a score model [13]. Shi et al. have established a classification and regression tree model including creatinine, bilirubin, prothrombin time, and leukocytes in a Chinese cohort [16]. However, highly specific diagnosis at high sensitivity also seems to be a challenge in these models [13,14,15,16].

Among the relevant discriminatory parameters identified by the Random Forest and the Lasso model, the use of PPI has previously been described as risk factor for SBP [17,18,19], suggesting the prudent use of PPI in patients with liver cirrhosis. From a pathophysiological point of view, an increase in small intestinal bacterial overgrowth, an alteration of intestinal microbial flora, impaired neutrophils’ function (in vitro), and delayed gastric emptying may be considered underlying factors, which are possibly exacerbated by the lower metabolism of omeprazole and pantoprazole in patients with liver cirrhosis [20]. The double-blind, placebo-controlled trial ‘Stop of Proton-pump Inhibitor Treatment in Patients With Liver Cirrhosis’ might provide further guidance [21]. Furthermore, intake of non-selective beta blockers leading to β1- and β2-adrenergic blockade, i.e., propranolol or carvedilol in our study, seems to be protective for the occurrence of infected ascites in the Lasso regression model. This observation supports previous findings [22]. This reduced risk for developing SBP may be attributed to a lower hepatic vein pressure gradient, reduced bacterial translocation by a decreased splanchnic blood flow and consequent less intestinal mucosa oedema and congestion [23], faster intestinal transit [24,25], and improved immune system performance due to inhibition of the stress-related cyclic adenosine monophosphate-protein kinase A pathway [25]. However, in the presence of SBP, non-selective beta blockers may be contraindicated due to renal insufficiency [26]. In addition, acute upper gastrointestinal bleeding is associated with an increased risk of infected ascites, as confirmed in this study, and antibiotic administration is recommended to prevent clinically relevant bacterial infections such as SBP [5,23].

To our knowledge, our study is the only one to date using a machine learning approach with >30 clinical features from a multicentre cohort that enables physicians to spare patients a potential abdominal puncture with very high negative predictive value. However, this study has certain limitations. Early diagnosis of infected ascites is crucial, and delayed diagnostic paracentesis in SBP is associated with increased in-hospital mortality [4]. The model values must be interpreted with caution in each case because the negative predictive value of the score model depends on the prevalence of infected ascites, which differs across countries with varying incomes [1]. The retrospective study design is a further limitation of the study and therefore, the model needs prospective multicentre evaluation. External validation of the performance of the models still needs to be conducted, in addition to the internal validation that has already been completed.

In summary, out of 34 clinical and laboratory features, the 11 most important discriminatory features were selected to establish a point-score model for differentiating infected from non-infected ascites in patients with from liver cirrhosis. Due to its high negative predictive value, this model helps physicians identify patients not requiring abdominocentesis, as supported by the low risk for infected ascites. However, the positive predictive value of our model is relatively low; thus, diagnosis of infected ascites without abdominocentesis should not be applied. 

## 4. Materials and Methods

### 4.1. Study Population

**Figure 2:**
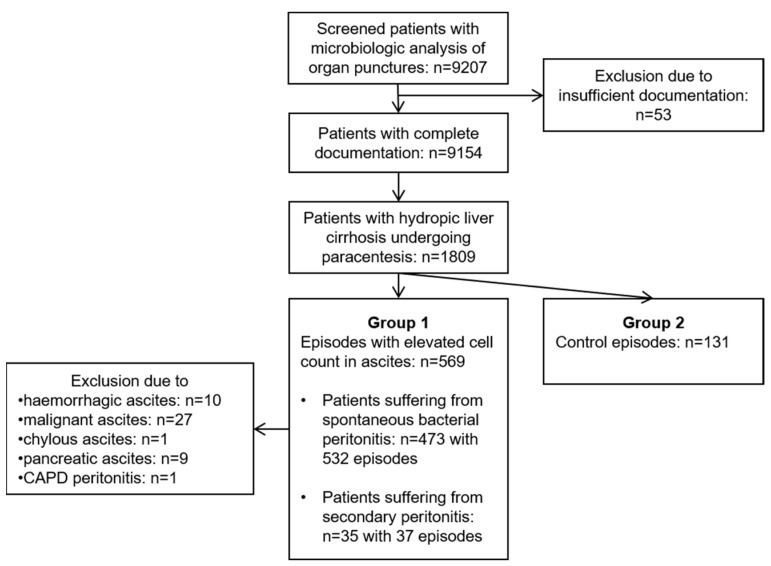
Flow chart of the screening and eligibility process of the study. An elevated cell count in ascites (group A) was defined by ≥250 polymorphonuclear leukocytes/mm^3^ and/or a leukocyte count ≥500/mm^3^. CAPD, continuous ambulatory peritoneal dialysis.

This multi-centre retrospective study was conducted in three tertiary care hospitals in Germany (University Hospital rechts der Isar of the Technical University of Munich, München Klinik gGmbH Bogenhausen, RoMed Klinikum Rosenheim). The screening and eligibility process is depicted in Figure 2. The HyBASE (Cymed, Bochum, Germany) microbiological database was used for identifying patients who underwent organ punctures between 2006 and 2020. Hospitalized patients were included in the study if they were ≥18 years old and had undergone abdominocentesis due to hydropic decompensated liver cirrhosis. The study patients were assigned to either group A (infected ascites) or group B (control group; non-infected ascites). Group A included all episodes with elevated cell count in ascites (n = 569), in line with the common definition of SBP by ≥250 polymorphonuclear leukocytes/mm^3^ and/or leukocyte count ≥500/mm^3^. SecP was defined as peritoneal infection secondary to intra-abdominal lesions (n = 32) or SecP following intra-abdominal surgery (n = 5). We excluded episodes with the following ascitic aetiology from group A: haemorrhagic, malignant, pancreatic, tuberculous, chylous ascites, and continuous ambulatory peritoneal dialysis peritonitis (CAPD). The control group (group B, n = 125 patients with 131 episodes) was formed through consecutive selection of individual episodes without an elevated cell count in ascites.

### 4.2. Assessment of Predictors for SBP and SecP

The Child–Pugh score, a model for end-stage liver disease with serum sodium (MELD-Na) score, and the acute-on-chronic liver failure (ACLF) score [27] were assessed for all the episodes. The definitions of organ failure were slightly modified according to CLIF-C ACLF criteria [28] as follows: serum creatinine ≥ 2 mg/dL; bilirubin > 12 mg/dL; INR ≥ 2.5; encephalopathy, and therapy with vasopressors or mechanic ventilation (intubation).

### 4.3. Ethical Statement

This study was approved by the Ethics Committee of the Technical University of Munich, School of Medicine, University Hospital rechts der Isar, Munich, Germany (approval no. 201/19 S-SR,), and was conducted in accordance with the Declaration of Helsinki. The institutional review board waived the requirement for written informed consent due to the retrospective nature of the study (World Health Organization trial registration number: DRKS00017728). 

### 4.4. Statistical Analysis

Laboratory blood features with more than 30% missing values were excluded from this study (pH, procalcitonin, lactate, lactate dehydrogenase, and albumin). Episodes were counted as independent if the patients were discharged from the hospital for at least two weeks without relapse. The distribution of continuous variables is described by the median and range, while categorical data are presented as absolute and relative frequencies. Group comparisons were performed using Fisher’s exact test or Pearson’s Chi-squared test on qualitative variables and Wilcoxon’s rank-sum test for quantitative variables. A conditional Random Forest model (using the parameter settings ‘minsplit’ = 9, ‘minbucket’ = 3 and ‘ntree’ = 1000) was used to rank the importance of predictors for diagnosis by internally validated (out-of-bag) permutation accuracy importance measures [29]. With n = 569 episodes in group A, a sample size calculation showed that a Mann–Whitney-U test at a two-sided 5% significance level achieves a power of 90% to even detect a weak area under the receiver operating characteristics curve (ROC) of 0.60 when 130 episodes are provided by group B. The least absolute shrinkage and selection operator (Lasso) regression model was applied to obtain an additive score model for dichotomous predictors [30]. Both models were fit and internally validated with established cut-off values for dichotomisation for the Lasso regression model applied to the ACLF score > 0 and Child-Pugh score > 9; all other optimal cut-off values were computed by maximized statistics [30]. The parameter shrinkage of the Lasso model was guided by the maximum area under the curve (AUC) value according to the ‘1se’ rule and using five-fold cross-validation. The resulting model coefficients were divided by the smallest coefficient and rounded to the next integer to obtain an additive point-score model. Fagan’s nomograms depict the post-test probability according to the Lasso regression model for detection of infected ascites. Statistical hypothesis testing was performed using two-sided exploratory significance levels of 0.05. All analyses and the web application were conducted in R version 4.0.3 (R Foundation for Statistical Computing, Vienna, Austria).

## Figures and Tables

**Table 1:** Lasso Score model. The main score and simplified score model to differentiate SBP/SecP from non-infected ascites in patients with hydropic decompensated liver cirrhosis. The probability of infected ascites increases with every score point. The ideal cut-off values of the main score model and the simplified score are >72 and >23.

Measurement	Score Points	Simplified Model
CRP > 4.2 mg/dL	+87	+26
Previous hydropic decompensation	+60	+17
White blood cell counts > 11.49 G/L	+52	+16
Organ failure *	+45	+14
Fever	+39	+13
Acute gastrointestinal bleeding	+31	+8
PPI medication	+26	+8
Previous SBP	+8	+5
Charlson Comorbidity Index > 6	+6	-
No propranolol or carvedilol medication	+1	+2
MELD-Na score > 24.9	+1	-
Child-Pugh class C	-	+1

* Occurrence of any of the following organ failures: serum creatinine ≥ 2 mg/dL; bilirubin > 12 mg/dL; INR ≥ 2.5; encephalopathy, therapy with vasopressors, mechanic ventilation. Abbreviations: CRP, C-reactive protein; PPI, proton pump inhibitors; SBP, spontaneous bacterial peritonitis; MELD-Na model of end-stage liver disease with serum sodium.

## Data Availability

The raw data are not publicly available as they contain extensive information that could compromise the privacy of research participants, but they are available from the corresponding author upon reasonable request.

## Supplementary Materials

The following supporting information can be downloaded at: https://www.mdpi.com/article/10.3390/antibiotics11111610/s1, Figure S1: Fagan’s nomograms for post-test probability of infected ascites; Table S1: Univariable comparison of group A and B.

## References

[1] Tay P.W.L., Xiao J., Tan D.J.H., Ng C., Lye Y.N., Lim W.H., Teo V.X.Y., Heng R.R.Y., Yeow M.W.X., Lum L.H.W. (2021). An Epidemiological Meta-Analysis on the Worldwide Prevalence, Resistance, and Outcomes of Spontaneous Bacterial Peritonitis in Cirrhosis. Front. Med..

[2] Soriano G., Castellote J., Álvarez C., Girbau A., Gordillo J., Baliellas C., Casas M., Pons C., Román E.M., Maisterra S. (2010). Secondary bacterial peritonitis in cirrhosis: A retrospective study of clinical and analytical characteristics, diagnosis and management. J. Hepatol..

[3] Wiest R., Schoelmerich J. (2009). Secondary peritonitis in cirrhosis: “Oil in fire”. J. Hepatol..

[4] Kim J.J., Tsukamoto M.M., Mathur A.K., Ghomri Y.M., Hou L., Sheibani S., A Runyon B. (2014). Delayed Paracentesis Is Associated with Increased In-Hospital Mortality in Patients with Spontaneous Bacterial Peritonitis. Am. J. Gastroenterol..

[5] Angeli P., Bernardi M., Villanueva C., Francoz C., Mookerjee R.P., Trebicka J., Krag A., Laleman W., Gines P. (2018). EASL Clinical Practice Guidelines for the management of patients with decompensated cirrhosis. J. Hepatol..

[6] European Association for the Study of the Liver (2010). EASL clinical practice guidelines on the management of ascites, spontaneous bacterial peritonitis, and hepatorenal syndrome in cirrhosis. J. Hepatol..

[7] Aithal G.P., Palaniyappan N., China L., Härmälä S., Macken L., Ryan J.M., Wilkes E.A., Moore K., Leithead J.A., Hayes P.C. (2021). Guidelines on the management of ascites in cirrhosis. Gut.

[8] Pache I., Bilodeau M. (2005). Severe haemorrhage following abdominal paracentesis for ascites in patients with liver disease. Aliment. Pharmacol. Ther..

[9] Kurup A.N., Lekah A., Reardon S.T., Schmit G.D., McDonald J.S., Carter R.E., Kamath P.S., Callstrom M.R., Atwell T.D. (2015). Bleeding Rate for Ultrasound-Guided Paracentesis in Thrombocytopenic Patients. J. Ultrasound Med..

[10] Lin S., Wang M., Zhu Y., Dong J., Weng Z., Shao L., Chen J., Jiang J. (2015). Hemorrhagic Complications Following Abdominal Paracentesis in Acute on Chronic Liver Failure. Medicine.

[11] Würstle S., Hapfelmeier A., Karapetyan S., Studen F., Isaakidou A., Schneider T., Schmid R.M., von Delius S., Gundling F., Triebelhorn J. (2022). Web Application. A Novel Machine Learning-Based Point-Score Model as a Non-Invasive Decision-Making Tool for Identifying Infected Ascites in Patients with Hydropic Decompensated Liver Cirrhosis: A Retrospective Multicentre Study. https://bookerar.shinyapps.io/Infected_ascites/.

[12] Alaniz C., Regal R.E. (2009). Spontaneous bacterial peritonitis: A review of treatment options. P T..

[13] Abdel-Razik A., Mousa N., Abdel-Aziz M., Elsherbiny W., Zakaria S., Shabana W., Abed S., Elhelaly R., Elzehery R., Eldars W. (2019). Mansoura simple scoring system for prediction of spontaneous bacterial peritonitis: Lesson learnt. Eur. J. Gastroenterol. Hepatol..

[14] Wehmeyer M.H., Krohm S., Kastein F., Lohse A.W., Lüth S. (2014). Prediction of spontaneous bacterial peritonitis in cirrhotic ascites by a simple scoring system. Scand. J. Gastroenterol..

[15] Metwally K., Fouad T., Assem M., Abdelsameea E., Yousery M. (2018). Predictors of Spontaneous Bacterial Peritonitis in Patients with Cirrhotic Ascites. J. Clin. Transl. Hepatol..

[16] Shi K.-Q., Fan Y.-C., Ying L., Lin X.-F., Song M., Li L.-F., Yu X.-Y., Chen Y.-P., Zheng M.-H. (2011). Risk stratification of spontaneous bacterial peritonitis in cirrhosis with ascites based on classification and regression tree analysis. Mol. Biol. Rep..

[17] Min Y.W., Lim K.S., Min B.-H., Gwak G.-Y., Paik Y.H., Choi M.S., Lee J.H., Kim J.J., Koh K.C., Paik S.W. (2014). Proton pump inhibitor use significantly increases the risk of spontaneous bacterial peritonitis in 1965 patients with cirrhosis and ascites: A propensity score matched cohort study. Aliment. Pharmacol. Ther..

[18] Dam G., Vilstrup H., Watson H., Jepsen P. (2016). Proton pump inhibitors as a risk factor for hepatic encephalopathy and spontaneous bacterial peritonitis in patients with cirrhosis with ascites. Hepatology.

[19] Elzouki A.-N., Neffati N., Rasoul F.A., Abdallah A., Othman M., Waness A. (2018). Increased Risk of Spontaneous Bacterial Peritonitis in Cirrhotic Patients Using Proton Pump Inhibitors. GE-Port. J. Gastroenterol..

[20] Ratelle M., Perreault S., Villeneuve J.-P., Tremblay L. (2014). Association Between Proton Pump Inhibitor Use and Spontaneous Bacterial Peritonitis in Cirrhotic Patients with Ascites. Can. J. Gastroenterol. Hepatol..

[21] Lohse A.W., Kluwe J., Wehmeyer M.H., Horvatits T. Stop of Proton-Pump Inhibitor Treatment in Patients with Liver Cirrhosis—A Double-blind, Placebo-controlled Trial (STOPPIT). ClinicalTrials.gov Identifier, NCT04448028. ClinicalTrials.gov 2020. NCT04448028.

[22] Leithead J.A., Rajoriya N., Tehami N., Hodson J., Gunson B.K., Tripathi D., Ferguson J.W. (2015). Non-selective β-blockers are associated with improved survival in patients with ascites listed for liver transplantation. Gut.

[23] Thalheimer U., Triantos C.K., Samonakis D.N., Patch D., Burroughs A.K. (2005). Infection, coagulation, and variceal bleeding in cirrhosis. Gut.

[24] Pérez-Paramo M., Muñoz J., Albillos A., Freile I., Portero F., Santos M., Ortiz-Berrocal J. (2000). Effect of propranolol on the factors promoting bacterial translocation in cirrhotic rats with ascites. Hepatology.

[25] Senzolo M., Cholongitas E., Burra P., Leandro G., Thalheimer U., Patch D., Burroughs A.K. (2009). Beta-Blockers protect against spontaneous bacterial peritonitis in cirrhotic patients: A meta-analysis. Liver Int..

[26] Mandorfer M., Bota S., Schwabl P., Bucsics T., Pfisterer N., Kruzik M., Hagmann M., Blacky A., Ferlitsch A., Sieghart W. (2014). Nonselective β Blockers Increase Risk for Hepatorenal Syndrome and Death in Patients with Cirrhosis and Spontaneous Bacterial Peritonitis. Gastroenterology.

[27] Moreau R., Jalan R., Gines P., Pavesi M., Angeli P., Cordoba J., Durand F., Gustot T., Saliba F., Domenicali M. (2013). Acute-on-Chronic Liver Failure Is a Distinct Syndrome That Develops in Patients with Acute Decompensation of Cirrhosis. Gastroenterology.

[28] CLIF-C-ACFL Calculator European Foundation for the Study of Chronic Liver Failure 2022. https://www.efclif.com/scientific-activity/score-calculators/clif-c-aclf.

[29] Hapfelmeier A., Hornung R., Haller B. (2022). Sequential Permutation Testing of Random Forest Variable Importance Measures. arXiv.

[30] Hothorn T., Lausen B. (2003). On the exact distribution of maximally selected rank statistics. Comput. Stat. Data Anal..

