# Supplementary material for: A Novel Machine Learning-Based Point-Score Model as a Non-Invasive Decision-Making Tool for Identifying Infected Ascites in Patients with Hydropic Decompensated Liver Cirrhosis: A Retrospective Multicentre Study
Source: Antibiotics (Basel). 2022 Nov 12;11(11):1610. doi: 10.3390/antibiotics11111610 (PMC9686825; doi:10.3390/antibiotics11111610)
Supplement: Supplementary file 1 [file antibiotics-11-01610-s001.zip › Figure S1.pdf]

Article

A Novel Machine Learning-Based Point-Score Model as a Non-Invasive Decision-Making Tool for Identifying Infected Ascites in Patients with Hydropic Decompensated Liver Cirrhosis: A Retrospective Multicentre Study

Figure S1: Fagan's nomograms for post-test probability of infected ascites.

- A Post-test probability of SBP or SecP for a point-score model >72 (full model)
- B Post-test probability of SBP or SecP for a point-score model ≤72 (full model)
- C Post-test probability of SBP or SecP for a point-score model >72 (simplified model)
- D Post-test probability of SBP or SecP for a point-score model ≤72 (simplified model)

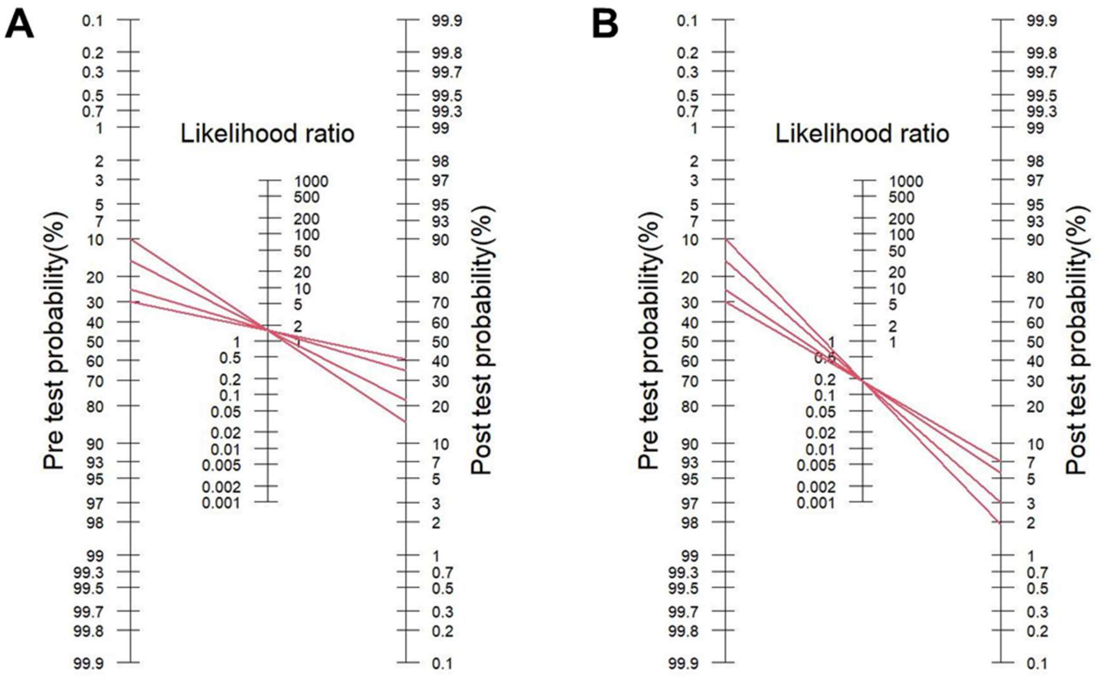

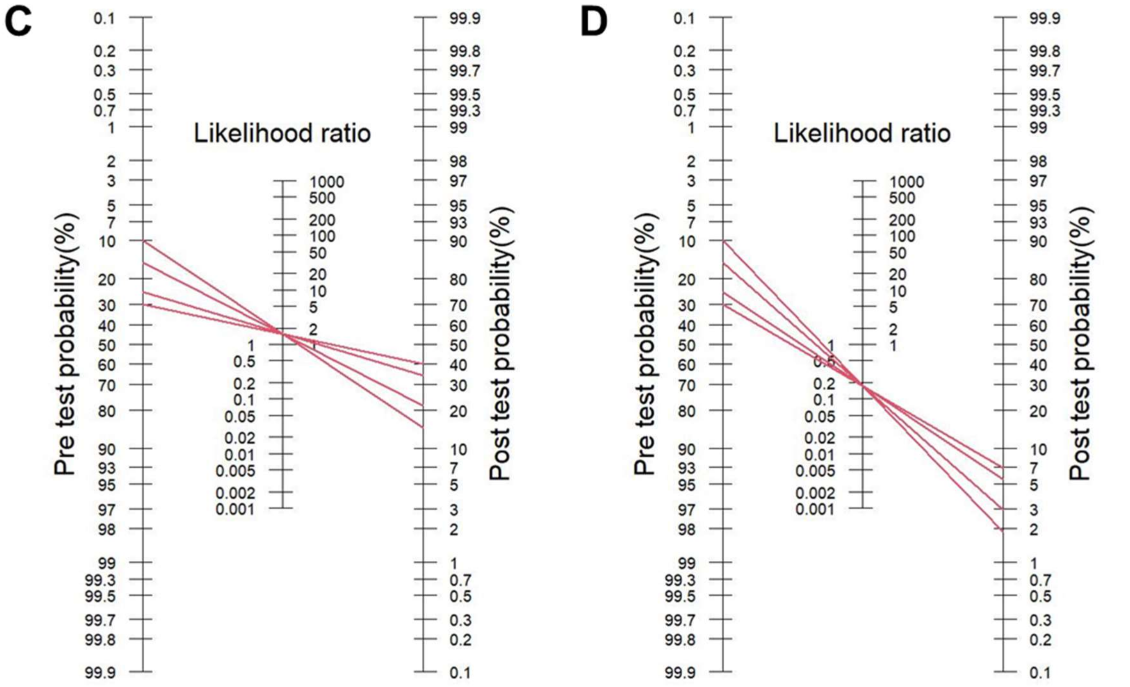

C
Likelihood ratio
Pre test probability(%)
Post test probability(%)
0.1 0.2 0.3 0.5 0.7 1 2 3 5 7 10 20 30 40 50 60 70 80 90 93 95 97 98 99 99.3 99.5 99.7 99.8 99.9
1000 500 200 100 50 20 10 5 2 1 0.5 0.2 0.1 0.05 0.02 0.01 0.005 0.002 0.001
99.9 99.8 99.7 99.5 99.3 99 98 97 95 93 90 80 70 60 50 40 30 20 10 7 5 3 2 1 0.7 0.5 0.3 0.2 0.1
D
Likelihood ratio
Pre test probability(%)
Post test probability(%)
0.1 0.2 0.3 0.5 0.7 1 2 3 5 7 10 20 30 40 50 60 70 80 90 93 95 97 98 99 99.3 99.5 99.7 99.8 99.9
1000 500 200 100 50 20 10 5 2 1 0.5 0.2 0.1 0.05 0.02 0.01 0.005 0.002 0.001
99.9 99.8 99.7 99.5 99.3 99 98 97 95 93 90 80 70 60 50 40 30 20 10 7 5 3 2 1 0.7 0.5 0.3 0.2 0.1
