# Supplementary material for: A Novel Machine Learning-Based Point-Score Model as a Non-Invasive Decision-Making Tool for Identifying Infected Ascites in Patients with Hydropic Decompensated Liver Cirrhosis: A Retrospective Multicentre Study
Source: Antibiotics (Basel). 2022 Nov 12;11(11):1610. doi: 10.3390/antibiotics11111610 (PMC9686825; doi:10.3390/antibiotics11111610)
Supplement: Supplementary file 1 [file antibiotics-11-01610-s001.zip › Table S1.pdf]

Article

A Novel Machine Learning-Based Point-Score Model as a Non-Invasive Decision-Making Tool for Identifying Infected Ascites in Patients with Hydropic Decompensated Liver Cirrhosis: A Retrospective Multicentre Study

Table S1: Univariable comparison of group A and B. Features are displayed as relative frequency in % (absolute frequency) or median (range). Creatinine was adjusted for dialysis based on the calculation of the MELD-Na score. ACLF score, acute-on-chronic liver failure score; ICU, Intensive Care Unit; MELD-Na, model for end-stage liver disease with serum sodium; NA, not available; SBP, spontaneous bacterial peritonitis.

Features	Group A (n=569)	Group B (n=131)	P-value
**Clinical features**			
Age	63 (23-92)	62 (31-91)	0.523
Previous SBP episodes	16.8% (94/559)	3.1% (4/131)	<0.001
Previous SBP episodes <3 months	16.8% (94/561)	3.1% (4/131)	<0.001
Previous hydropic decompensation of liver cirrhosis	75.4% (364/483)	56.3% (72/128)	<0.001
Alcohol-associated cause of liver cirrhosis	74.5% (424/569)	79.4% (104/131)	0.291
Previous upper gastrointestinal bleeding <3 months	6.3% (35/554)	3.8% (5/131)	0.086
Acute upper gastrointestinal bleeding	12.1% (69/569)	5.3% (7/131)	0.036
Previous hepatic encephalopathy	4.7% (27/569)	5.3% (7/131)	0.951
Acute hepatic encephalopathy	37.1% (211/569)	13.0% (17/131)	<0.001
Ongoing chronic alcohol abuse at admission	33.8% (181/538)	51.2% (63/123)	0.001
Fever at admission	35.2% (172/488)	3.8% (5/128)	<0.001
First diagnosis/ascites puncture on ICU	17.8% (101/567)	3.2% (4/126	<0.001

Pleural effusion	40.9% (226/553)	23.8% (30/126)	0.001
Esophageal varices	62.8% (329/524)	68.6% (81/118)	0.371
Cancer disease	21.9% (124/566; 3 NA)	15.3% (20/131)	0.198
Diabetes	32.7% (186/569)	26.7% (35/131)	0.222
Chronic renal failure	22.2% (126/568)	19.1% (25/131)	0.578
Previous liver or kidney transplantation	2.6% (15/569)	0% (0/131)	0.087
**Medication**			
Lactulose	60.6% (345/569)	56.5% (74/131)	0.439
Proton-pump inhibitors	84.9% (483/569)	76.3% (100/131)	0.025
Non-selective beta-blockers	38.1% (217/569)	54.2% (71/131)	0.001
Immunosuppressive therapy	14.1% (78/490)	3.8% (5/131)	<0.001
**Laboratory features in blood**			
Sodium in serum (mmol/L)	134 (109-160; 14 NA)	135 (119-148; 1 NA)	0.384
Creatinine in serum (mg/dL)	1.8 (0.9-4.0; 10 NA)	1.1 (1.0-4.0; 6 NA)	<0.001
C-reactive protein in serum (mg/dL)	6.3 (0.1 - 34.3; 89 NA)	1.7 (0.03 – 10.2; 15 NA)	<0.001
Bilirubin in serum (mg/dL)	3.5 (0.2-46.4; 79 NA)	2.1 (0.4-30.6; 12 NA)	<0.001
Glucose in serum (mg/dL)	119 (9-408; 151 NA)	116 (71-328; 28 NA)	0.920

Leukocytes in blood (G/L)	9.5 (1.2 – 41.0; 6 NA)	6.2 (2.2 – 20.4)	<0.001
Platelets in blood (G/L)	113 (4-676; 6 NA)	128 (20-420; 1 NA)	0.034
Internationalized normalized ratio	1.5 (0.9-7.1; 47 NA)	1.4 (1.0-3.1; 4 NA)	<0.001
**Clinical scores**			
ACLF score >0	63.8% (363/569)	22.1% (29/131)	<0.001
0	36.2% (206/569)	77.9% (102/131)	
1	18.8% (107/569)	7.6% (10/131)	
2	18.1% (103/569)	9.2% (12/131)	
3	26.9% (153/569)	5.3% (7/131)	
Child-Pugh score	10 (7-15)	9 (7-13)	<0.001
MELD-Na score	26.5 (6.2 - 40.0; 110 NA)	19.0 (7.5 - 39.0; 15 NA)	<0.001
Charlson Comorbidity Index	7 (3-20)	6 (3-14)	<0.001
